# Herbal Medicines—Are They Effective and Safe during Pregnancy?

**DOI:** 10.3390/pharmaceutics14010171

**Published:** 2022-01-12

**Authors:** Beata Sarecka-Hujar, Beata Szulc-Musioł

**Affiliations:** 1 Department of Basic Biomedical Science, Faculty of Pharmaceutical Sciences in Sosnowiec, Medical University of Silesia in Katowice, Kasztanowa Str. 3, 41-200 Sosnowiec, Poland; 2 Department of Pharmaceutical Technology, Faculty of Pharmaceutical Sciences in Sosnowiec, Medical University of Silesia in Katowice, Kasztanowa Str. 3, 41-200 Sosnowiec, Poland; bszulc@sum.edu.pl

**Keywords:** herbs, herbal medicines, ginger, cranberry, garlic acid, *Ginkgo biloba*, *Echinacea purpurea*

## Abstract

Since the teratogenicity of Thalidomide has been proven, herbal products are more commonly used in pregnancy to not only relieve morning sickness but also to fight infections. These products are frequently considered as natural and therefore harmless. However, herbs contain a number of active substances that, when used during pregnancy, can affect the development of the fetus. Often, pregnant women do not consult the usage of herbal medicines with a physician. The access to these products is easy and treatment of certain ailments with the use of herbs is common in many countries. The aim of the present literature review was to discuss available data regarding the efficacy and safety of cranberry, chamomile, *Echinacea purpurea*, garlic, ginger, *Ginkgo biloba*, and peppermint, which are used to counteract the most common ailments during pregnancy, i.e., infections and pregnancy-related ailments (e.g., nausea and vomiting, dizziness, and headache). Analysis of available data showed that ginger is one of the most extensively analyzed herbal remedies. The dose of ginger below 1000 mg per day may help to relief *hypereremesis gravidarum*, and such an amount of ginger did not increase frequency of adverse effects for either woman or developing fetus. Data regarding other herbs are most often heterogeneous and give conflicting results with no clear conclusions. However, all herbal products should be used with a special caution in pregnancy. Further high-quality human studies should be determined to confirm the safe doses of herbal products which could be used by pregnant or breast-feeding women.

## 1. Introduction

For centuries, herbal products have been used to treat various diseases in different regions of the world. A number of herbal medicines contain antimicrobial, anti-inflammatory, and immunomodulatory properties and may be used in treatments e.g., of infections. In general, the use of herbs during pregnancy is not advisable due to their possible impact on the fetal development. Numerous experimental studies were earlier conducted to analyze the effect of extracts from various herbs on mice or rats fetuses. However, available results are often contradictory. Some of the studies showed morphological and toxic effects of herbal extracts on animal progeny while others did not [1,2,3]. In the study by Lewicki et al. [1], pregnant and lactating mice were fed with aqueous and hydro-alcoholic extracts of *Rhodiola kirilowii* as well as epigallocatechin (EGC), which is an antiangiogenic compound of *Rhodiola* extracts. Authors found some morphometric anomalies in the kidney structure in the offspring of mothers fed with hydro-alcoholic extracts of *Rhodiola kirilowii* or EGC. On the contrary, application of *Scutellaria baicalensis* extract demonstrated no significant effects in fetal parameters in three groups of mice receiving 2, 8 or 32 g of extract/kg/day. However, a high dose of *Scutellaria baicalensis* was suggested to have some potential maternal toxicity because absolute liver and kidneys weights were significantly increased in the group receiving 32 g of this herbal extract/kg/day compared to controls [2]. Similarly, in the case of *Boehmeria nivea,* no embryotoxicity in mice was observed but significantly lower viability of embryonic stem cells was demonstrated [3].

Since the teratogenicity of Thalidomide has been proven, herbal medicines are more commonly used during pregnancy to relieve pregnancy-related symptoms and help fight infections, among others. Herbs are frequently considered as natural and therefore harmless. However, these products were suggested to influence the CYP superfamily, which is responsible for 65–80% of all CYP-mediated drug metabolism [4]. Some of the herbs which were observed to be used by pregnant women show potent inhibition into the following: CYP1A2 (black elderberry, ginger, and horsetail), CYP2D6 (fennel and raspberry leaf), and CYP3A4 (fennel and raspberry leaf) [5].

Pregnant women usually do not consult the use of herbs with a doctor or pharmacist [6]. Data demonstrated that almost 95% of Ethiopian pregnant women did not consult the usage of herbal medicines with the doctor or nurse [7]. In Norway, only less than 12% of pregnant women were recommended to use herbs by healthcare personnel [8]. Such a situation results from the fact that future mothers are not aware that herbs contain active substances that can cause miscarriage, premature birth, uterine contractions, or injury to the fetus. On the other hand, the clinicians do not always have sufficient knowledge on the safety of using herbal medicines during pregnancy and while breastfeeding.

Previously, it was demonstrated that a large number of women used many types of herbal medicines for different purposes during their pregnancies [9]. The use of herbal medicines by pregnant women differs between Europe and USA, reaching from 27% to 57% or even more in Europe and from 10% to 73% in the USA [10]. In a study of 400 pregnant Norwegian women, the prevalence of herbs use was 36% and surveyed women indicated to using nearly 250 herbal products containing 46 different herbs [8]. Conversely, in southern Italy, 81% of pregnant women indicated the use of at least one herbal product during pregnancy [11]. Interestingly, the frequencies of Australian women using herbal remedies during pregnancy and while breastfeeding were lower (14% and 10%, respectively) [12]. Previously, complementary and alternative medicine (CAM), which includes phytotherapy, was demonstrated to be used by women between 31–40 years of age with higher educational levels, and they also used CAM in their earlier pregnancies [13]. Nevertheless, the problem of the use of herbs during pregnancy is noteworthy, because it concerns both the developing fetus and the woman.

The aim of the present literature review was to discuss available data regarding the efficacy and safety of the herbal products (cranberry, chamomile, *Echinacea purpurea*, garlic, ginger, *Ginkgo biloba*, and peppermint) used to counteract the most common ailments during pregnancy. The results of surveys and clinical trials concerning the effects of the selected herbal medicines on developing fetus as well as the results of the experimental studies performed in animals were discussed in the present review.

## 2. Methodology

We searched PubMed, Google Scholar, and Embase using the following keywords in different combinations: “pregnancy”, “pregnant women”, “labor”, “herbs”, “herbal medicines”, “cranberry”, “chamomile”, “Echinacea purpurea”, “garlic”, “ginger”, “Ginkgo biloba”, and “peppermint” (last search November 2021). In the present literature review, we focused our attention on herbs that are frequently used for the most common ailments during pregnancy: (1) herbs used to counteract urinary infections and upper respiratory tract infections, i.e., cranberry, *Echinacea purpurea*, and garlic; (2) herbs used to counteract some pregnancy-related symptoms (nausea and vomiting, dizziness, and headache), i.e., chamomile, ginger, *Ginkgo biloba*, and peppermint. We included experimental data on animals, as well as clinical trials and surveys on pregnant women. Finally, we discussed the results which we believed were the most interesting or relevant.

## 3. Herbal Medicines Used to Counteract Infections during Pregnancy

### 3.1. Cranberry (Vaccinium macrocarpon)

*Vaccinium macrocarpon* (American cranberry; also known as large cranberry) belongs to the *Ericaceae* family. It is native to North America, growing across the United States and Canada, and its fruits are large, spherical red berries. In contrast, wild growing swamp cranberry (*Vaccinium oxycoccus*) is characterized by smaller fruits. Cranberries contain the highest total phenolic amount and are characterized by a highest total antioxidant capacity compared to other fruits [14,15]. The antioxidant activity is related to the presence of three main classes of flavonoids: anthocyanins, flavonols, and flavan-3-ols, which play an important role in the prevention of bacterial infections, especially urinary tract infections (UTI) [16,17]. Previously it was demonstrated that cranberry extracts reduce C-reactive protein (CRP) and proinflammatory interleukins but increase synthesis of nitric oxide [17]. In addition, oral administration of anthocyanin from bilberry (*Vaccinium myrtillus*) resulted in endothelium-dependent vasodilation through the activation of the NO-cGMP signaling pathway in hypercholesterolemic individuals [18]. Cranberries or products containing cranberry extracts are often recommended for the treatment of UTI, also during pregnancy.

In an experimental study by Bałan et al. [19], pregnant and lactating mice were fed with American cranberry extract of a daily dose of 0.88 mg. The authors observed some differences in the progeny’s spleens, more CD19+ and CD8+ lymphocytes in the cytometry analysis of spleen cells, higher serum concentration of VEGF and bFGF, as well as an increase in the diameter of kidneys glomeruli in the offspring from the cranberry group compared to the control group. However, no differences were seen in the response to immunization by red blood cells of sheep, and no abnormalities in creatinine and urea serum level were also observed in progeny of mice fed with cranberries [19].

As to studies on humans, in the study by Wing et al. [20], none of the 14 pregnant women receiving cranberry capsules to treat URI had antepartum complications, i.e., fetal malformation, intrauterine fetal growth restriction, oligohydramnios, or polyhydramnios. Neonatal outcomes were similar between the study and placebo groups, however 1-min Apgar score < 7 was more frequent in the cranberry group (21%) than in the placebo group (0%). Similarly, in a large cohort of Norwegian pregnant women, in which 1.3% of women had used cranberry, no increased risk of congenital malformations after its usage was also observed [21]. Another Norwegian survey among pregnant women showed that 6% of future mothers used cranberry for treatment of UTI [22]. Statistically significant differences in urinary interleukin-6 level was demonstrated when comparing pregnant women consuming cranberry juice and placebo group (receiving beverage without cranberry) [23]. Conversely, according to four Brazilian medical societies, there is weak evidence to support the use of cranberries and cranberry-derived dietary supplements in the treatment of lower urinary tract infections in pregnant and non-pregnant women [24]. Table 1 presents the characteristics and findings of selected studies, evaluating the use of cranberries during pregnancy, both in animals and humans.

### 3.2. Echinacea purpureae L. (Moench.)

The *Echinacea purpurea* L. (Moench.) is a native medicinal plant from North America used by many Indian tribes for curing toothache and throat pain but also against rabies and infectious diseases [25]. Herbal products containing Echinacea are one of the most commonly used in the USA. *Echinacea purpurea* comprises many chemical compounds, i.e., water-soluble polysaccharides (arabinoxylan and arabinogalactan types), caffeic acid derivatives (cichoric, caftaric, and chlorogenic acids), isobutylamides, flavonoids (quercetin and kaempferol), glycoproteins, essential oils, amines, and polyacetylenes. Due to such a rich composition, Echinacea shows various activities, i.e., anti-inflammatory, antifungal, antiviral, antibacterial, as well as immunomodulatory. The immunological effect of Echinacea was analyzed in many studies. Previously, it was demonstrated that in rats fed with an Echinacea-rich diet, the production of IgA, IgG, and IgM was significantly increased in comparison to control rats [26]. In turn, in mice treated with *Echinacea purpurea,* the splenic lymphocytes were significantly more resistant to apoptosis [27]. Polysaccharides and isobutylamides are responsible for the immunomodulatory effect of Echinacea. These compounds can activate the immune system’s cells and in turn increase the amount of leukocytes and the intensity of phagocytosis of macrophages and granulocytes. By inhibiting the formation of mediators of an inflammatory state, i.e., prostaglandins and leukotrienes, they also induce secretion of pro- and anti-inflammatory cytokines [28].

Previously, the possible influence of *Echinacea purpurea* extracts on angiogenic activity and tissue VEGF and bFGF production of mice fetuses was examined [29]. The authors demonstrated that Echinacea may interfere with embryonal angiogenesis. Another study showed that feeding pregnant mice with *Echinacea purpurea* decreased the number of viable fetuses [30].

Surprisingly, in a double-blind, placebo-controlled crossover study of 40 healthy males, the immunologic stimuli of *Echinacea purpurea* was unconfirmed [31]. The authors did not observe enhanced phagocytic activity of polymorphonuclear leukocytes or monocytes in the group of subjects receiving commercially available Echinacea compared to the placebo group [31]. However, in another study performed in healthy volunteers, it was found that *Echinacea purpurea* can inhibit cytochrome CYP1A2 and selectively modulate the catalytic activity of CYP3A4 at hepatic and intestinal sites [32].

According to the EMA monograph, Echinacea is not recommended in children below 12 years of age due to insufficiently documented safety [33]. Similarly, the use in pregnancy and lactation is not recommended unless advised by a doctor. The topical use of *Echinacea purpurea* is acceptable, omitting the breast area. In contrast, the German commission E Monographs indicate no restrictions on the use of *Echinacea purpurea* formulations during pregnancy or lactation, with the exception of the parenteral application [34]. As with other herbal products, there is also little information on the safety of using *Echinacea purpurea* during pregnancy, which is probably safe in recommended doses [35]. A study conducted in Norway showed no relation between using Echinacea and risk of developmental defects or adverse side effects in pregnant women [21]. The studies by Gallo et al. [36,37] showed that the reported use of Echinacea during organogenesis did not increase the risk of major or minor malformations. Over 200 women using Echinacea products during pregnancy were analyzed and compared to the group of pregnant women not using Echinacea. Among examined women, 13 spontaneous abortions, 6 major malformations were observed, while in the control group—7 spontaneous abortions, and 7 major malformations with no statistical differences [37]. According to Perri et al. [35], oral consumption of Echinacea during the first trimester did not increase the risk for major malformations and recommended doses of Echinacea were safe during pregnancy and lactation. Holst et al. [38] identified twenty clinical trials analyzing efficacy of various Echinacea preparations in different populations. However, for obvious reasons, such trials are not performed in pregnant women. The findings of selected studies evaluating the use of *Echinacea purpurea* during pregnancy both in animals and humans are presented in Table 2.

### 3.3. Garlic (Allium sativum)

Garlic contains several sulfur-containing compounds such as alliin, diallylsulfides, and allicin, which show antimicrobial activity, especially against *Escherichia coli*, *Staphylococcus aureus*, and *Escherichia faecalis*, but also against *Candida* spp. and *Trichomonas vaginalis* [39,40]. It was also suggested that use of garlic may prevent group B *Streptococcus* (GBS) disease in newborns and in consequence may decrease the costs of the antibiotics which are administered during the labor [41]. *Garlic* may reduce oxidative stress and blood pressure as well as inhibit platelet aggregation, which suggests its role in prevention of pre-eclampsia. However, the available data in the topic are often contradictory [42,43].

In animal model, garlic (administered with a dose of 100 mg/kg body weight (bw) for three weeks) was found to influence the amelioration of lipid parameters of both pregnant *Wistar* dams and their offspring as a result of its antioxidant activity [44]. Significant effects of garlic and ascorbic acid, administered to pregnant *Wistar* rats, in reduction of lead level in blood and brain were also shown [45]. The findings suggested that garlic may protect against lead-induced neuronal cell apoptosis in the developing hippocampus of rat pups [45]. The beneficial effects of this natural antioxidant in protecting against lead toxicity were also noted in other studies [46,47]. Co-administration of lead (at a dose of 160 and 320 mg/kg bw) with garlic extract (250 mg/kg bw) to pregnant rats resulted in reductions in lead levels not only in the mother’s blood but also in the cerebellum, placenta, and fetal brain [47]. In immunohistochemical staining, a reduction in the number of Purkinje cells in the cerebellum was observed in the group of animals exposed to lead. Additionally, degenerating pyknotic cells and vacuolization in the Purkinje cell layer were found. Garlic treatment attenuated the histopathological changes caused by lead administration in both the female and fetal cerebellum.

According to the results by Hsu et al. [48], garlic oil supplementation during pregnancy and lactation protects adult rat offspring against hypertension induced by perinatal high-fat diet. Garlic oil, as a precursor of H2S, was shown to increase the mRNA expression and activity of H2S-producing enzymes in the kidneys of offspring. Furthermore, it was observed that garlic oil therapy resulted in stimulation of *Lactobacillus* and *Bifidobacterium* growth and decrease of *Turicibacter* and *Staphylococcus genera* in the gut microbiota [48].

The results of a randomized, double-blind, placebo-controlled trial conducted in 44 pregnant women showed decreased levels of serum C-reactive protein and increased plasma glutathione in women obtaining 400 mg garlic in one tablet for 9 weeks compared to the placebo group [49]. In a large cohort of pregnant Norwegian women, garlic was demonstrated to decrease the risk of spontaneous preterm delivery, which may result from microbial infection during pregnancy [50].

A Saudi study demonstrated that 56% of the 297 pregnant women surveyed used herbal medicine, and 15.4% of them used garlic and 13.4% ginger [51]. Most women (i.e., 82%) relied on informal sources to use herbal supplements during pregnancy [51]. Garlic, together with ginger, was previously found to be the most frequently used herbal product by pregnant women from Ethiopia [6,52,53,54]. Comparatively, a study based on 350 women from the West Bank of Palestine demonstrated that 77.1% of them were taking herbs during pregnancy and lactation [55]. The survey results showed that the most commonly used plants during pregnancy were peppermint, sage, and anise, while during lactation: cinnamon, anise, peppermint, and sage. On the other hand, garlic was not used because it changes the smell of breast milk. The authors also showed that sometimes there were differences between the traditional and scientific uses of herbal remedies by the women surveyed [55]. The results of selected studies regarding the use of garlic during pregnancy, both in animals and humans, are presented in Table 3.

## 4. Herbal Medicines Used to Counteract Some Pregnancy-Related Symptoms (e.g., Nausea and Vomiting, Dizziness and Headache, and Memory Loss)

### 4.1. Chamomile (Matricaria chamomilla L.)

Chamomile belongs to the Asteraceae family. For centuries, it was traditionally used to treat wounds, ulcers, eczema, or skin irritations, but also in the management of neuralgia, rheumatic pain, and eyes disorders. There are two different chamomile plants: German chamomile and Roman chamomile. The common German variety comes from the flower of *Matricaria recutita*, while the less common Roman variety comes from the flower of *Chamaemelum nobile.* The benefits of using chamomile are due to the fact that the essential oils and flower extracts derived from chamomile contain more than 120 chemical components, many of which are pharmacologically active. They include, among others: sesquiterpenes (e.g., bisabolol and farnesene), sesquiterpenelactones (e.g., chamazulene and matricin), flavonoids (e.g., apigenin and luteolin), and volatile oils [56,57,58]. Studies have shown that chamomile may be used as a mild digestive relaxant to treat, among others, indigestion, motion sickness, nausea, and vomiting [59].

Modares et al. [60] conducted a randomized, placebo-controlled, triple-blind study, which was based on 105 pregnant women with mild to moderate vomiting and nausea. Participants took 500 mg ginger root powder, or 500 mg dried German chamomile flower or starch capsules (500 mg, placebo group) for 1 week. The authors observed that Rhodes index score (vomiting and depressed reflexes) in the chamomile group was significantly lower than in the placebo group (*p* < 0.05) [60].

Chamomile was reported to be one of the most common herbs used by pregnant women from north-east Italy [61]. A total of 44% of analyzed women used it through oral or topical route to counteract anxiety, digestive problems, and stretch marks. The authors observed that 21.6% of regular users of chamomile during pregnancy showed higher frequency of threatening miscarriages and preterm labors compared to non-users [61]. In addition, in two neonates after a regular maternal consumption of chamomile, a cardiac malformation (presumably related to Down syndrome), and an enlarged kidney were diagnosed.

On the other hand, no effect of chamomile use during the last two trimesters on premature birth was reported in a large Canadian population-based study [62]. The authors observed similar frequency of women using chamomile in both a group who had premature delivery and in a term delivery group.

In Pakistan, chamomile is used by traditional birth attendants to relieve labor pain. However, a study by Zafar et al. [63] found no significant difference in pain relief during labor between women using Chamomile and women receiving Pentazocine. An Iranian study analyzed the effectiveness of chamomile in inducing labor in women with a gestational age of 40 weeks or more, cephalic presentation, an absence of uterine contraction, as well as a cervical Bishop score of less than four [64]. Women in the study group received capsules with chamomile extract (500 mg) for 7 days (6 capsules a day). The authors observed that the onset of labor was significantly faster (*p* = 0.003) in the chamomile group than in the placebo group [65]. In another study, Heidari-Fard et al. [65] applied chamomile aromatherapy to women in labor at 4 cm dilatation and continued it until the end of delivery. It has been shown that the chemocline odor significantly reduces the intensity of contractions in dilatation of 5–7 cm in the chamomile group compared to the control group.

A study by Silva et al. [66] reported a case of a 29-year-old Caucasian Portuguese woman who noticed an abundant amount of milk and breast enlargement 4–6 h after drinking chamomile tea. From 3 to 6 months after delivery, the patient drank about 1.5 to 2 liters of chamomile infusion a day. As the analyzed woman observed, her milk production increased from about 60 mL to about 90 mL during this time. Although the mechanism of chamomile’s galactogogue effect remains unclear, it has been assumed that chamomile may elicit its estrogenic activity by inhibition of CYP 1A1, 1A2, and 3A4 by its bioactive compounds such as apigenin, luteolin, quercetin, and caffeic acid [67,68].

Table 4 shows the results of selected studies analyzing the usage of chamomile during pregnancy, both in animals and humans.

### 4.2. Ginger (Zingiber officinale Rosc.)

Rhizome is a therapeutic part of ginger (Zingiberis Rhizoma). It contains two active complexes: Gingerol, a phenolic compound giving a sharp taste, and Shogaol [69]. Gingerol shows antiemetic activity and Shogaol acts as a 5-HT antagonist on serotonin receptors in the ileum. Other active compounds of ginger are as follows: non-volatile substances (resins), essential oil (e.g., aromatic hydrocarbons sesquiterpenes, zingiberen, kurkumen, and β-bisabolen), as well as lipids and glycolipids [70]. Ginger works through many interesting mechanisms, i.e., increases gastrointestinal transport with the effects characteristic to prokinetic antiemetic drugs [71], and in consequence ingested food has less time to cause upper gastrointestinal problems such as vomiting. Ginger also suppresses vasopressin, which may cause nausea in high levels, and inhibits stomach activity, which can also result in nausea [69]. The anticoagulant effect of ginger and the interactions between ginger and anticoagulant drugs were also observed [72], suggesting its complete avoidance during the anticoagulant therapy. However, Jiang et al. [73] found no effect of ginkgo and ginger on pharmacokinetic and pharmacodynamics of warfarin in 12 healthy male subjects as well as no effect on blood clotting status or platelet aggregation. Ginger is “generally regarded as safe” by the Food and Drug Administration (FDA) in the United States. On the other hand, in Finland, the Finnish Food Safety Authority does not recommend ginger products (i.e., tea or food supplements containing ginger) during pregnancy due to the lack of known safe consumption limits [74]. Ginger is used most commonly to relieve nausea and vomiting during the first trimester of the pregnancy.

In animal model, no maternal and developmental toxicity was observed after administration of zingiber extract at daily doses of up to 1000 mg/kg bw to the pregnant rats (6 to 15 days of gestation) [75]. The study by ElMazoudy and Attia demonstrated that ginger at doses of 1000 and 2000 mg/kg bw/day caused maternal toxicity, with increased mortality and a significant decrease in body weight gain in female mice [76]. The authors also showed that a dose of 2000 mg/kg in females significantly reduced the number of live fetuses and increased fetal death and resorption, but no adverse effects were observed for the doses of 250 and 500 mg/kg. The daily administration of ginger extract (at a dose of 200 mg/kg bw) to pregnant rats during the organogenesis phase of gestation, i.e., from 6th to 15th day of gestation, 1 h after gabapentin injection, minimized the brain damage in rat fetuses caused by this antiepileptic drug [77]. In addition, the immuno-histochemical investigation found that co-administration of ginger after gabapentin injection resulted in a significant decrease in the expression of the pro-apoptotic marker Caspase-3 and an increase in the expression of the anti-apoptotic marker Bcl-2 in fetal rat brains. Another study by El-Borm et al. [78] showed that ginger extract (200 mg/kg) significantly ameliorated the pathological changes in fetal cardiac tissue induced by labetalol, a drug used to treat maternal hypertension during pregnancy.

Ginger is undoubtedly the most analyzed herbal remedy used in pregnancy. Most recently, a Swiss cross-sectional survey, comparing the usage of herbal medicines during pregnancy in women with and without mental disorders or symptoms (MDS), showed that among 272 respondents, the ginger (49.2%), raspberry leaf (42.7%), bryophyllum (37.8%), chamomile (27.2%), lavender (22%), and iron-rich herbs (12.3%) were the most frequently taken herbal medicines [79]. The vast majority of pregnant women participating in the study rarely chose synthetic psychoactive medications for the treatment of MDS, but were more likely to use pharmaceutical herbal products (valerian, lavender, and bryophyllum).

The Canadian study demonstrated that women who used ginger against nausea and vomiting found it only moderately effective [80]. However, meta-analysis performed on a large group of almost 1300 pregnant women showed that ginger significantly improved the symptoms of nausea and showed some improvement in reducing the number of vomiting episodes compared to the placebo group [81]. The effectiveness of ginger in relieving nausea and vomiting was also compared to vitamin B6 in the study by Sharifzadeh et al. [82]. A total of three groups of pregnant women between 6 and 16 weeks of pregnancy were administered with ginger, vitamin B6, and placebo, respectively. The authors found that ginger is comparable with vitamin B6 in the treatment of mild to moderate nausea and vomiting during pregnancy as well as being more effective than the placebo [82]. Interestingly, in the study by Smith et al. [83], the frequency of spontaneous abortions was lower in the group of women who were ingesting ginger compared to those ingesting vitamin B6. On the other hand, in the double-blind, randomized, placebo-controlled trial by Willetts et al. [84], 120 women in less than 20 weeks pregnant randomly received wax sealed capsules containing 125 mg of ginger extract or soya bean as placebo. The authors observed that the nausea experience score was significantly lower for the ginger extract group than in the placebo group, while no significant effect was observed on vomiting. In turn, in the case of *hyperemesis gravidarum*, a double-blind, randomized trial showed that ginger significantly (*p* = 0.035) relieved symptoms in women receiving 1 g of ginger compared to pregnant women receiving placebo [85].

Tianthong and Phupong [86] investigated in a randomized, double-blind, placebo-controlled trial the efficacy of ginger capsules in the prevention of abdominal distention in 89 post cesarean section women. They demonstrated that the incidence of postoperative abdominal distention was not different between the ginger and the placebo groups, but the severity of abdominal distention on the fourth day after operation was significantly lower in the ginger group than the placebo group. In the ginger group, the number of women who could eat in this condition was higher than in the placebo group (59.6% vs. 43.8%, *p* = 0.035). In addition, defecation was faster in the ginger group.

Hajimoosayi et al. [87] reported that ginger tablets taken for 6 weeks by women with gestational diabetes mellitus with impaired glucose tolerance test significantly reduced fast blood glucose, serum insulin, and Homeostasis Model Assessment index compared to the placebo group. In addition, there was no significant reduction of the serum blood glucose 2 h post-prandial in both groups, which, according to the authors, could be due to the use of the same diet in both groups. However, ginger intake was related to a shorter gestational age as well as to a lower circumference of the newborn’s skull in Italian pregnant women compared to those who did not use ginger [11]. A cross-sectional study conducted among women in early pregnancy or postpartum women from Scotland identified eight herbal products (aloe, chamomile, cranberry, fish oil, ginger, ginseng, grapefruit, and sage) that may cause interactions with concomitant prescription drugs [88]. Of the 34 potential interactions, one between ginger and nifedipine was reported as potentially major.

Ginger was also observed to enlarge milk production after delivery. Women from Thailand receiving 500 mg dried ginger capsules twice daily have significantly higher milk volume than the placebo group (191.0 ± 71.2 mL/day and 135.0 ± 61.5 mL/day, respectively) [89]. However, the authors demonstrated that the seventh day milk volume in women taking ginger capsules did not differ from the milk in women from the placebo group.

The results of selected studies regarding the use of ginger during pregnancy, both in animals and humans, are presented in Table 5.

### 4.3. Gingko biloba L.

*Ginkgo biloba* L. is a tree belonging to the ginseng family which originated from south-east regions of China. Extracts from *Ginkgo biloba* are one of most widely used as well as best studied herbal products. These complex mixtures contain numerous components, mostly including flavonoids (e.g., quercetin) and terpene lactones and in addition bioflavonoids, ginkgolic acids, and ginkgotoxin, showing a vitamin B6-similar structure [90,91]. The *Ginkgo biloba* extract has many advantages but when applied at high doses it may be toxic to the cells, i.e., it may increase human red blood cells fragility, influence cellular morphology, and induce glutathione consumption [92]. Ginkgo has no specific uses during pregnancy or breastfeeding; however, it may be a component of herbal mixtures or teas. It may be commonly used as an antioxidant or a vasodilator to increase cerebral and peripheral perfusion. The most obvious reason of usage of Ginkgo is to improve memory but also to manage headaches.

In 2016, extract from leaves of *Ginkgo biloba* was classified as a possible human carcinogen (Group 2B) by the International Agency for Research on Cancer [93]. Previously, *Ginkgo biloba* extract was demonstrated to indicate carcinogenic activity in mice due to increased incidence of hepatocellular carcinoma and hepatoblastoma [94].

In animal models, the possible impact of *Ginkgo biloba* extract on fetus is well documented. No toxic effect on the *Wistar* dams and no fetal malformations were observed after using *Ginkgo biloba* extract [95]. However, earlier *Ginkgo biloba* was found to have reproductive toxicity in the mouse model. Zehra et al. [96] observed that fetuses from mice who received higher doses of Ginkgo extract (i.e., 100 mg/kg/day) showed increased frequency of malformations including round shaped eye and orbits, syndactyly, and malformed pinnae, nostrils, lips, and jaws. The study concerning possible effects of 6-gingerol from *Ginger*, Ginkgolide A, and Ginkgolide B from *Gingko biloba* and Ginsenoside Rg1 from *Ginseng* was performed in the chick embryonic heart micromass and Mouse D3 embryonic stem cells with analysis of the following cell aspects: alteration in contractility, cell viability, and cell protein content [97]. The results showed that herbs used in the first trimester of pregnancy might not be safe for fetal development. *Ginkgo biloba* extract may show a neurotrophic effect, which was demonstrated in fetuses of pregnant rats administered with 100 or 300 mg/kg/day *Ginkgo biloba* extract for 5 days [98]. The increased number of hippocampal neurons as well as altered expression of 160 or 187 genes in the hippocampi of female or male fetuses, respectively, were observed in this study [98].

Chen et al. [99] analyzed a perinatal hypoxic-ischemic on neonatal male rats. In the study, it was observed that administration of Ginkgolide B 30 minutes before ischemia induction resulted in a decrease in the level of interleukin (IL-1β and IL-18) and inhibition of NLRP3 inflammasome activation, which corresponded to a reduced infarct volume and an alleviation of cerebral edema in the study group.

Data on the usage of Ginkgo extract during pregnancy in women are scarce. A study by Holst et al. [100] identified Ginkgo as one of the herbs used by Swedish women during pregnancy, mostly to improve circulation and cognitive functioning. The study by Petty et al. [101] demonstrated correlation between the use of herbal supplements and the appearance of colchicine in placental blood. Significant levels of colchicine (3 µg/tablet) were found by the authors in the commercially available Ginkgo biloba formulations [101]. Colchicine shows anti-inflammatory, anti-metastatic, and teratogenic activities, which were previously found to be fatal in high doses [102]. However, data from Israel did not report colchicine as a human teratogen because the rate of major congenital anomalies was comparable between the pregnancies exposed on colchicine and those who were not exposed. Furthermore, no cytogenetic anomalies were observed in the colchicine group [103]. Ginkgo should be avoided especially around labor because it could prolong bleeding time due to its possible anti-platelet properties. Table 6 demonstrates characteristics and findings of selected studies regarding the use of *Ginkgo biloba* during pregnancy, both in animals and humans.

### 4.4. Peppermint (Mentha piperita)

Peppermint leaves and oil contain, among others: menthol (35–45%), menthone, menthyl acetate, neomenthol, isomenthone, limonene, rosmaric acid, and flavonoids [104]. It shows a spasmolytic effect on the smooth muscle of the digestive tract but also antiviral, antimicrobial, or diuretic activities [105,106,107,108,109]. Both peppermint oil and menthol can positively affect nausea and vomiting by acting on the 5-HT(3) receptor ion–channel complex, probably by binding to a modulatory site, which is different from the site of serotonin binding [110]. According to Hines et al. [111], inhalation aromatherapy with peppermint compared to placebo may cause little or no difference in the severity of postoperative nausea and vomiting. Postoperative nausea and vomiting are common postoperative complications, the causes of which may include anesthesia, anxiety, and stress [112]. In a single-blind, randomized, controlled trial, it was shown that inhalation of 0.2 mL of 10% and 30% peppermint essential oil in each of the study groups significantly reduced the severity of nausea after the intervention [112]. In many clinical trials, inhalation of peppermint oil has been shown as effective in reducing the perceived severity of postoperative nausea and, according to the authors, should be considered as a first-line treatment that can be used quickly [113,114,115,116,117]. In addition, peppermint oil aromatherapy offers a rapid onset of action, ease of administration, and the absence of any serious side effects [118].

Peppermint is often used to relieve nausea and vomiting in pregnancy. Experimental studies showed that peppermint may not have any teratogenic effect in mice fetuses [119]. The authors demonstrated that when using hydroalcoholic extract of *Mentha piperita* at doses 600 and 1200 mg/kg/day continuously during embryonic period, no serious congenital malformations or delayed bone ossification were found in the fetuses [119].

Several studies regarding the impact of peppermint on pregnancy in women were previously published [120,121,122,123,124,125,126,127,128]. Many studies pointed to the possibility of using aromatherapy, one of the types of complementary medicine, as a care option for pregnant women to minimize nausea and vomiting in early pregnancy. In this regard, a randomized clinical trial involving 90 pregnant women showed a significant reduction in the severity of nausea and vomiting on the second, third, and fourth days of inhalation aromatherapy with lemon and peppermint in the study group compared with baseline assessment, before the intervention [120]. The study by Joulaeerada et al. [121] showed that inhalation with peppermint oils by pregnant women decreased the severity of nausea and vomiting, but this reduction was not statistically significant compared to the placebo group, which used sweet almond oil. Similar effects of inhalation aromatherapy with peppermint essential oil treatment were reported by Pasha et al. [122]. They observed that the intensity of nausea and vomiting in the peppermint aromatherapy group was higher than in the saline group, but it was not significant.

In turn, peppermint oil was observed to be effective in reducing the severity of pruritus, which concerns up to 8% of pregnant women [123]. Menthol, the main peppermint component, decreases itching caused by histamine by cooling the skin. However, when used in an excessive way, it may induce uterine bleeding in early pregnancy [123]. In the study by Kıssal et al. [124], peppermint was found to be commonly used against cold-influenza in 1st and 2nd trimesters of pregnancy.

In the literature, peppermint used in normal doses has been classified as safe for pregnant women [125]. Study results from eight cross-sectional studies (2729 participants) from seven Asian countries showed that approximately 47% of women had used at least one herbal medicine during a previous pregnancy. Of the 33 herbs, peppermint was mentioned the most (22.8% of women) [125]. In addition, a cross-sectional study of 320 Iranian women found that 48.4% of them used herbal medicines during pregnancy, of which sour orange (30.97%), peppermint (19.81%), and borage (19.46%) were the most commonly indicated [126]. Pregnant women with secondary education and from higher socio-economic classes were more likely to use herbs.

Amzajerdi et al. [127] observed in pregnant women that after 7 days of using the mint flavor, the mean score of nausea and vomiting in the study group was significantly lower than in the control group, although in both groups on the last day of the intervention, the mean score of nausea and vomiting decreased significantly. In another study, Ghani et al. [128] observed a significant decrease in nausea and vomiting episodes on the third day of mixed essential oils (peppermint and lavender) inhalation in early pregnancy.

In turn, a Canadian study found that peppermint was not related to the preterm delivery [62]. The authors observed comparable frequency of women using peppermint during the whole pregnancy in the group of over 600 women who delivered before 37 weeks of gestation as in the group of over 2500 women with term delivery (2.09% vs. 1.79%, respectively). The summary of the main findings of selected studies evaluating the use of peppermint during pregnancy both in animals and humans is demonstrated in Table 7.

## 5. Conclusions

Herbal medicines are used during pregnancy worldwide with a different prevalence depending on traditions and geographical diversity of the region. In the Ethiopian population, rural residency, illiteracy, and low average monthly income significantly predicted the usage of herbs in pregnancy [6,78]. The nature of scientific approach to phytotherapy has changed over the years. The “CAMbrella” project, funded by the European Union and implemented between 2010 and 2012, was one of the first attempts of a comprehensive approach to the problem of CAM, with phytotherapy included [129,130]. Previously, active herbal compounds were tested mainly in vitro and in vivo, while currently, clinical settings have started being more common. Nevertheless, there are still little clinical data analyzing herbal products in pregnant women and when they do exist, they are carried out on a small number of participants and therefore offer statistically weak results.

Our review of available data regarding the usage of herbal remedies to counteract the ailments during pregnancy shows that ginger is one of the most extensively analyzed. In pregnant women with *hypereremesis gravidarum*, taking 1 g of ginger per day resulted in a significantly higher mean relief score compared to the placebo group [85]. In addition, ginger was observed to have comparable effect to vitamin B6 [83]. Experimental data on animal models showed that ginger in a dose below 1000 mg/kg did not cause any toxicity but doses of 1000 mg/kg or 2000 mg/kg resulted in maternal toxicity [76]. Data on pregnant women also did not report increased frequency of adverse effects from use of ginger in a dose below 1000 mg. Therefore, its usage during pregnancy seems to be safe both for future mother and for developing fetus.

Data regarding other herbs are most often heterogeneous and give conflicting results with no clear conclusions. Of these, garlic intake during pregnancy may significantly reduce the risk of both early and late spontaneous preterm delivery [50]. Several data also reported the beneficial effects of inhalation with peppermint oil to relieve nausea and vomiting [120,121,127]. However, there is still not enough evidence to prove the effectiveness of cranberries, Echinacea, Ginkgo biloba, chamomile, or peppermint in pregnant women. This uncertainty is also evident in the guidance of drug approval authorities, as ginger is “generally regarded as safe” by the FDA, while the Finnish Food Safety Authority does not recommend ginger products during pregnancy due to the lack of known safe consumption limits.

The main limitations of the present review were the quantity and quality of the available data, which creates difficulty in drawing obvious conclusions. Some symptoms relief has been shown with the use of certain herbs, but these data may not be transferable to different disease severities. Undoubtedly, all herbal products should be used with a special caution in pregnancy, as safe consumption limits are lacking. Further methodologically reliable studies on larger groups of pregnant women are needed to confirm the safe doses of herbal products, which could be used by pregnant or breastfeeding women.

## Figures and Tables

**Table 1:** Characteristics of the studies on the use of cranberries during pregnancy both in animals and humans.

**Experimental Studies**						
**Reference**	**Animal Species**	**Treatment**	**Sample Size (*N*)**		**Adverse Effects**	**Main Results**
Bałan et al. [19]	Mice inbred females of Balb/c strain	44 mg cranberry extract/kg b.m./day, since copulatory plug, up to the 28-th day after delivery.	18		The morphological disorder of the spleen.	Significantly (*p* < 0.05) larger glomerular diameter in the offspring from cranberry group than in the control group, more CD19+ and CD8+ lymphocytes in the cytometry analysis, higher serum concentration of VEGF and bFGF.
**Human Studies**						
**Reference**	**Cohort Allocation**	**Study Design**	**Treatment**	**Sample Size (*N*)**	**Adverse Effects**	**Main Results**
Wing et al. [20]	USA	A pilot randomized, placebo-controlled study.	Two cranberry capsules, at a gestational age of less than 16 weeks until delivery.	33	1st min Apgar score < 7 was more frequent in the cranberry group than in the placebo group (21% vs. 0%, respectively).	Seven cases of asymptomatic bacteriuria were observed in 5 patients: 2 of 24 in the cranberry group and 3 of 25 in the placebo group. There were no cases of cystitis or pyelonephritis.
Nordeng et al. [22]	Norway	Interview using a structured questionnaire.	Herbal medicines (most common: ginger, iron-rich herbs, Echinacea, and cranberry).	600	Use of raspberry leaves prior to delivery was associated with an increased risk of caesarean delivery.	A total of 39.7% of women used herbal drugs during pregnancy, of which 86.3% used conventional drugs. A significant association between the use of iron-rich herbs and high birthweight.
Wing et al. [23]	USA	A randomized, controlled pilot investigation.	A total of 27% cranberry juice cocktail two times daily.	27	None observed.	Cranberry juice cocktail consumption significantly reduced urinary IL-6 in the study group compared to placebo group.

**Table 2:** Characteristics of the studies on the use of *Echinacea purpurea* during pregnancy both in animals and humans.

**Experimental Studies**						
**Reference**	**Animal Species**	**Treatment**		**Sample Size (*N*)**	**Adverse Effects**	**Main Results**
Barcz et al. [29]	Mice	A total of 0.6 mg of Echinacea purpurea extract from the 1st day of fertilization until the 18th day of pregnancy. Tablets: Immunal Forte (LEK, Ljubljana, Slovenia), Echinapur (Herbapol Poznañ, Poznan, Poland) and Esberitox (Schaper & Brümmer GmbH & Co. KG, Salzgitter, Germany).		18	The morphological disorder of the spleen.	Significantly (*p* < 0.05) larger glomerular diameter in the in the offspring from cranberry group than in the control group; more CD19+ and CD8+ lymphocytes in the cytometry analysis, higher serum concentration of VEGF and bFGF.
**Human Studies**						
**Reference**	**Cohort Allocation**	**Study Design**	**Treatment**	**Sample Size (*N*)**	**Adverse Effects**	**Main Results**
Heitmann et al. [21]	Norway	Cohort Study	Herbal products containing Echinacea for use during pregnancy.	68,522	No increased risk of malformations or adverse effects in pregnancy.	Among 68,522 women, 363 (0.5%) reported the use of Echinacea during pregnancy. There was a prevalence of 1.5% of major malformations among the women who had used Echinacea compared with 2.6% in non-users.
Gallo et al. [37]	Canada	A prospective study	A total of 114 (58%) of 198 women used capsule or tablet preparations, or both, of Echinacea (250 to 1000 mg/d) ,76 (38%) of the subjects used tinctures (5 to 30 drops per day). Duration of use: 5 to 7 days.	206	Rates of malformations between the study and control groups did not significantly differ.	A total of 112 women (54%) used Echinacea in the first trimester, with 17 (8%) exposed in all three trimesters. A total of 81% of respondents noticed an improvement in the symptoms of upper respiratory tract ailments.

**Table 3:** Characteristics of the studies analyzing the use of garlic during pregnancy both in animals and humans.

**Experimental Studies**						
**Reference**	**Animal Species**	**Treatment**		**Sample Size (*N*)**	**Adverse Effects**	**Main Results**
El-Sayyad et al. [44]	Rats	A total of 100 mg homogenate of garlic/kg bw for three weeks prior to onset of gestation as well as throughout the gestation period.		60	Not mentioned	*Allium sativum* supplementation to hypercholesterolemic pregnant rat decreased the incidence of abortion and abnormalities of the newborn, improved ossification, and ameliorated the histological picture of myocardial muscle fiber of their offspring.
Ebrahimzadeh-Bideskan et al. [45]	Rats	Lead-treated group: lead acetate in the drinking water (1500 ppm) Lead plus Garlic-treated group: to lead acetate in the drinking water (1500 ppm) + 1 mL Garlic juice (daily dose of 100 g/kg bw by gavage during pregnancy and lactation.		50	Not mentioned	Significantly reduced blood and brain lead levels in young rats in the Lead + Garlic group (*p* < 0.01) compared to lead-treated animals
						Significant decrease in the number of apoptotic cells in all subregions of the hippocampus (*p* < 0.05) in the Lead plus Garlic-treated group vs. in the lead-treated group.
Saleh et al. [47]	Rats	Two Pb-treated groups (exposed to 160 and 320 mg/kg bw of Pb, respectively). Two groups treated with both Pb and garlic (exposed to Pb as previous groups together with 250 mg/kg bw/day of garlic extract). Treatments: once a day from GD 1–20 of pregnancy.		40	Not mentioned	Immunohistochemical examination of the cerebellar cortex of rats in the low dose Pb-treated group showed irregular arrangement and wide separation between Purkinje cells and in high dose Pb-treated group, a decrease in the number of Purkinje cells was evident. Simultaneous administration of garlic extract + Pb resulted in a significant reduction in Pb levels in maternal blood and brain, placenta, and placental and fetal brain.
Hsu et al. [48]	Rats	Pregnant rats received either a normal diet (ND) or 58% high-fat diet. Garlic oil (GO) or vehicle was administered daily at 100 mg kg^−1^ day^−1^ during pregnancy and lactation. Male offspring were fed with the same diet as their dams until 16 weeks. Experimental groups (*N* = 8/group): ND, HF, ND + GO, and HF + GO.		32	Not mentioned	Maternal garlic oil therapy: - Prevented high-fat diet-induced hypertension in adult rat offspring; - Increased mRNA and activity of H2S-producing enzymes in offspring’s kidneys; - Increased growth stimulation of the Lactobacillus and Bifidobacterium.
**Human Studies**						
**Reference**	**Cohort Allocation**	**Study Design**	**Treatment**	**Sample Size (*N*)**	**Adverse Effects**	**Main Results**
Ziaei et al. [43]	Iran	Randomized, single-blind, placebo-controlled study.	Two tablets of garlic (800 mg/day) during the third trimester of pregnancy for 8 weeks. Each garlic tablet contained 1000 μg allicin, and ajoene.	100	Not mentioned	No significant difference between the two groups in the means of HDL, LDL, triglyceride, systolic and diastolic blood pressure, inhibition of platelet aggregation, and the mean arterial blood pressure. Significant difference in the means of total cholesterol (*p* = 0.038) and hypertension alone (*p* = 0.043).
Aalami-Harandi et al. [49]	Iran	Randomized, double-blind, placebo-controlled trial.	Participants received one garlic tablet (400 mg garlic and 1 mg allicin) once daily for 9 weeks.	44	No serious adverse reactions were reported.	A significant decrease in the level of high sensitivity CRP (*p* = 0.01) and an increase in the concentration of glutathione (GSH) in the plasma (*p* = 0.03). Serum lipid profiles, plasma total antioxidant capacity (TAC) levels, and pregnancy outcomes are similar in both groups.
Myhre et al. [50]	Norway	Population-based pregnancy cohort study.	Alliums (garlic, onion, leek, and spring onion) intake Garlic usage was classified as: low intake: ≤0.40 g and high intake: >0.40 g.	18,888	Not mentioned	Out of 18,888 deliveries, 950 (5.0%) spontaneous preterm deliveries were found. Garlic intake significantly reduces the risk of both early (OR: 0.47 (95% CI: 0.25; 0.89)) and late (OR: 0.83 (95% CI: 0.71, 0.98)) spontaneous preterm delivery.

**Table 4:** Characteristics of the studies on the use of chamomile during pregnancy in humans.

Human Studies						
Reference	Cohort Allocation	Study Design	Treatment	Sample Size (*N*)	Adverse Effects	Main Results
Moderes et al. [60]	Iran	Triple-blind randomized placebo-controlled trial.	1st group: ginger capsules (500 mg ginger root powder) 2nd group: chamomile capsules (500 mg dried chamomile flower) 3rd group: starch capsules (500 mg, placebo) twice a day for one week.	105	Chamomile group: skin allergy, ginger group: diarrhea and vomiting.	No significant difference between chamomile and ginger after one week of treatment. Significant difference between chamomile and placebo after one week of treatment.
Zafar et al. [63]	Pakistan	Double blind randomized controlled trial.	Anesthesia of the woman in the early stage of labor (3–6 cm): - Conventional group: intramuscular injection of Pentazocine (30 mg/mL); - Homeopathy group: three drops of 1 M solution chamomile; - Placebo group: dispensed of sugar pellets.	99	Not mentioned	No significant differences in pain were noticed between three groups (Chamomile, Pentazocine, and placebo).
Gholami et al. [64]	Iran	Double-blind clinical trial study.	A total of two capsules every 8 h for 7 days (1 capsule contained 500 mg chamomile extract).	80	Not mentioned	Gestational age at the time of delivery in the chamomile group was significantly shorter than the placebo group.
Heidari-Fard et al. [65]	Iran	Randomized clinical trial.	A total of two drops of chamomile essence was added to a gauze. Aromatherapy started during dilatation of 4 cm and continued to the end of delivery. Use of the essence was repeated every half an hour for three times in the range of determined dilatations.	130	Not mentioned	Duration of contractions and number of contractions of the first delivery were similar in chamomile and control groups. In dilatation of 5–7 cm, intensity of contractions in the intervention group was significantly (*p* = 0.004) lower than the control group.

**Table 5:** Characteristics of the studies analyzing the use of ginger during pregnancy both in animals and humans.

**Experimental Studies**						
**Reference**	**Animal Species**	**Treatment**		**Sample Size (*N*)**	**Adverse Effects**	**Main Results**
Weidner et al. [75]	Rats	EV.EXT 33, a patented *Zingiber officinale* extract 1st group: 100 mg/kg 2nd group: 333 mg/kg 3rd group: 1000 mg/kg Placebo: sesame oil Treatment: by oral gavage from days 6 to 15 of gestation.		22	No deaths or adverse reactions were observed	On day 21 of pregnancy, for a dose of 1000 mg/kg, no adverse effects for both maternal and developmental toxicity were observed.
ElMazoudy et al. [76]	Mice	Mice received ginger orally at 0, 250, 500, 1000 or 2000 mg/kg bw/day.		25 female mice/group (125 mice in total)	Doses of 1000 and 2000 mg/kg bw/day resulted in maternal toxicity	Mice treated with 2000 mg/kg bw/day displayed significant decreases in implantation sites. In this dose, ginger impaired the normal growth of corpus luteum because of progesterone insufficiency during early pregnancy.
Badawy et al. [77]	Rats	1st control group: 1 mL distilled water, intraperitoneally. 2nd ginger group: 200 mg/kg, per os 3rd GBP group: 162 mg/kg intraperitoneal injection 4th GBP + ginger group: intraperitoneal injection of GBP first followed by oral administration of ginger 1 h later. Treatment: from days 6 to 15 of gestation.		24 pregnant rats, 36 fetuses	Not mentioned	Co-administration of gabapentin (GBP) with ginger during pregnancy reduces the neurotoxicity of the antiepileptic drug.
El-Borm et al. [78]	Rats	1st control group: distilled water 2nd ginger group: 200 mg/kg, 3rd labetalol group: 300 mg/kg 4th labetalol + ginger group: oral injection of labetalol first followed by oral administration of ginger 1 h later. Treatment: from days 6 to 15 of gestation.		60	Not mentioned	Co-administration of ginger extract with labetalol during pregnancy significantly ameliorated labetalol-induced apoptosis as well as DNA damage, histological and ultrastructural changes in the cardiac tissue of rat fetuses.
**Human Studies**						
**Reference**	**Cohort Allocation**	**Study Design**	**Treatment**	**Sample Size (*N*)**	**Adverse Effects**	**Main Results**
Gantner et al. [79]	Switzerland	Cross-sectional survey.	Pharmaceutical herbal preparations for mild MDS treatment during pregnancy.	398	Well tolerability.	Out of 398 women, 272 used pharmaceutical herbal products, including ginger (49.2%), raspberry leaf (42.7%), bryophyte (37.8%), chamomile (27.2%), lavender (22%), and iron-rich herbs (12.3%). In the treatment of Mild Mental Disorders (MDS), pregnant women were more likely to choose herbal (mainly: St. John’s wort, hops, valerian, lavender, and bryophytes) than synthetic medications
Westfall et al. [80]	Canada	Semi-structured interviews.	Doses were not specified.	27	Not mentioned.	Of 27 women, 10 used herbal products to counteract nausea and vomiting. Moderate reduction in nausea and vomiting was observed.
Viljoen et al. [81]	South Africa	Randomized controlled trials.	Ginger intervention: fresh root, dried root, powder, tablets, capsules, liquid extract, and tea. Dose from 600 to 2500 mg ginger/day.	1278	Not pose a risk for side-effects or adverse events during pregnancy.	Nausea: significantly reduces the number of nausea episodes (MD 1.20, 95% CI 0.56–1.84, *p* = 0.0002) compared to placebo. Vomiting: no significant difference between the groups.
Sharifzadeh et al. [82]	Iran	Triple-blind clinical trial.	1st group: ginger capsules (500 mg) 2nd group: vitamin B6 capsules (40 mg) Treatment: twice daily for 4 days between 6 and 16 weeks of pregnancy.	77	Ginger showed no adverse effects on pregnancy outcome.	Ginger and vitamin B6 are more effective than placebo in reducing NVP (*p* = 0.039 and *p* = 0.007, respectively).
Smith et al. [83]	Australia	Randomized, controlled equivalence trial.	0.5 g of ginger or 75 mg of vitamin B6 daily for 3 weeks.	291	Women taking ginger reporting belching after ingestion.	Ginger was equivalent to vitamin B6 in reducing nausea (mean difference 0.2, 90% confidence interval (CI) −0.3, 0.8), retching (mean difference 0.3; 90% CI −0.0, 0.6), and vomiting (mean difference 0.5; 90% CI 0.0, 0.9).
Willetts et al. [84]	Australia	Randomized, controlled equivalence trial.	- the active treatment: 125 mg ginger extract (equivalent to 1.5 g of dried ginger) - the placebo: soya bean oil four times a day.	120	No adverse effects were observed.	The nausea experience score was significantly lower for the women taking ginger extract after the first day of treatment and this difference was present for each treatment day than for women from placebo group. No significant effect was observed on vomiting.
Fischer-Rasmussen et al. [85]	Denmark	Double-blind, randomized, cross-over trial.	1st group: ginger capsules (250 mg of powdered root ginger) 2nd group: placebo capsules (250 mg of lactose) Treatment: four times daily for 4 days, followed by a 2-day wash out before alternate treatment	30	No side effects were observed.	Mean relief scores highlighted greater relief of symptoms after ginger treatment compared to placebo (*p* = 0.035).
Hajimoosayi et al. [87]	Iran	Randomized, double-blind, placebo-controlled clinical trial.	Three tablets of ginger (1500 mg)/day, for six weeks.	70	Not mentioned.	Statistically significant differences in the levels of Fast Blood Sugar (*p* = 0.04), serum insulin (*p* = 0.01), and Homeostasis Model Assessment index (*p* = 0.05) compared to the placebo group.
Trabace et al. [11]	Italy	Retrospective observational study.	The most commonly used herbal products were chamomile, fennel, propolis, cranberry, lemon balm, ginger, valerian, and mallow.	600	Ginger intake resulted in a shorter gestational age and a smaller head circumference of newborns	A total of 81% of women consistently used at least one herbal product throughout their pregnancy. The most commonly used herbal products were chamomile, fennel, propolis, cranberry, lemon balm, ginger, valerian, and mallow.
McLay et al. [88]	United Kingdom	Cross-sectional survey.	Participants used prescribed medicines, or herbal or natural products, or both.	889	Potentially major interactions: ginger–nifedipine. Moderate interactions: ginger–metformin ginger–insulin ginger–aspirin.	A total of 44.9% of pregnant women ingested prescription drugs simultaneously with at least one herbal and natural products. Of this group, 12.7% of the women had 34 moderate herbal drug interactions, one assessed as potentially major (ginger and nifedipine) and one as minor (ondansetron and chamomile).

**Table 6:** Characteristics of the studies analyzing the use of *Ginkgo biloba* during pregnancy both in animals and humans.

**Experimental Studies**						
**Reference**	**Animal Species**	**Treatment**		**Sample Size (*N*)**	**Adverse Effects**	**Main Results**
Fernandes et al. [95]	Rats	Extract of *Ginkgo bilboa* in concentrations of 3.5, 7.0, and 14.0 mg/kg/day, from the 1st to the 8th day of pregnancy.		64	Not observed.	There was no significant difference between the number of live and dead fetuses, average fetal weight/offspring, and average placenta weight/offspring between experimental groups and control group.
Zehra et al. [96]	Mice	*Ginkgo biloba* extract 78, 100 mg/kg/day throughout the gestational period.		18	In the group receiving 100 mg/kg/day, reduced body weight and malformations were observed: round shape of the eye and orbits, malformed pinnae, nostrils, lips and jaws.	Fetuses from groups 78 mg/kg/day did not show any gross abnormalities. Not allowed in pregnancy, even for nutritional value.
**Human Studies**						
**Reference**	**Cohort Allocation**	**Study Design**	**Treatment**	**Sample Size (*N*)**	**Adverse Effects**	**Main Results**
Holst et al. [100]	Sweden	Registry-based study.	Herbal drugs reported used by Swedish women during early pregnancy.	787	The mother’s use of the examined herbal drugs was not significantly associated with any negative effects for the infant. According to the authors, too low a number of exposures does not allow to exclude the influence of herbal drugs on rare outcomes e.g., specific malformations.	Among 787 using herbal drugs during early pregnancy, four (0.5%) reported usage *Ginkgo biloba* to improve circulation and cognitive function.

**Table 7:** Characteristics of the studies analyzing the use of peppermint during pregnancy, both in animals and humans.

**Experimental Studies**						
**Referenc** **e**	**Animal Species**	**Treatment**		**Sample Size (*N*)**	**Adverse Effects**	**Main Results**
Golalipour et al. [119]	Balb/c Mice	Two experimental groups receiving 600 mg/kg/day and 1200 mg/kg/day orally of *Mentha piperita* extract and two control groups: one control group received normal saline orally by oral intubation. The other control group did not receive normal saline.		A total of 12 mice in experimental groups.	Not observed.	No signs of maternal toxicity due to *Mentha piperita* treatment.
**Human Studies**						
**Reference**	**Cohort Allocation**	**Study Design**	**Treatment**	**Sample Size (*N*)**	**Adverse Effects**	**Main Results**
Safajou et al. [120]	Iran	Double-blind, randomized clinical trial.	Inhalation with three drops of a solution containing 5% lemon essential oil and 5% mint essential oil when feeling nauseous for 4 days of treatment.	90	No side effects have been reported.	Nausea and vomiting scores; significantly greater on the second, third, and fourth days of intervention in peppermint and lemon group vs. in placebo group.
Joulaeerad et al. [121]	Iran	Single-blind clinical trial.	Inhalation aromatherapy with 10% peppermint essential oil for four days.	56	Not mentioned.	Before and during the four-day intervention period mean scores of NVP significantly decreased in both the study (peppermint essential oil) and placebo (sweet almond oil) groups.
Pasha et al. [122]	Iran	Double-blind clinical trial.	Inhalation 4 drops of mint oil for four consecutive nights before sleeping.	60	Not mentioned.	Mint oil aromatherapy has not been effective in reducing gestational nausea and vomiting.
Akhavan et al. [123]	Iran	Triple-blind clinical trial.	60 mL of peppermint oil 0.5% in sesame oil/twice a day for 2 weeks.	96	It has been shown that that peppermint oil did not create any special side effects on subjects.	The severity of itching was significantly reduced (*p* = 0.001) in peppermint group vs. placebo group.
Ahmed et al. [125]	Asian countries: Iran, Malaysia, Palestine, Iraq, Jordan, Oman, and Egypt	Eight cross-sectional studies.	Various pharmaceutical forms.	2729	Not shown any harmful effect to mother or fetus.	Studies in human pregnancy have shown no adverse effect of peppermint. Excessive dose can induce menstruation and cause miscarriage.
Abdollahi et al. [126]	Iran	Cross-sectional analytic study	Participants used of 20 herbs	320	Not tested.	A total of 48.4% of women used herbal medicines during pregnancy, of which 19.81% used peppermint.
Amzajerdi et al. [127]	Iran	Quasi-experimental interventional study.	Inhalation of four drops of pure mint oil twice a day for seven days of treatment.	66	Not tested.	After 7 days of intervention, the mean scores of Rhodes index and severity of nausea and vomiting in the intervention group were significantly (*p* < 0.001) lower than in the control group.
Ghani et al. [128]	Saudi Arabia	Randomized controlled trial.	Inhalation of mixed two perfumes of lavender and peppermint oils (2 drops of essential oil per 100 drops of carrier oil) twice a day, prior napping or sleeping.	101	Not mentioned.	On the third day of essential oils inhalation, significantly decreased (*p* < 0.0001) nausea and vomiting episodes in the study group compared with baseline. Significance increase in energy level associated with little decrease in fatigue score.

## Data Availability

Not Applicable.
